# RETRACTED: Hasanin et al. Exploration of Despair Eccentricities Based on Scale Metrics with Feature Sampling Using a Deep Learning Algorithm. *Diagnostics* 2022, *12*, 2844

**DOI:** 10.3390/diagnostics15182384

**Published:** 2025-09-19

**Authors:** Tawfiq Hasanin, Pravin R. Kshirsagar, Hariprasath Manoharan, Sandeep Singh Sengar, Shitharth Selvarajan, Suresh Chandra Satapathy

**Affiliations:** 1 Department of Information Systems, Faculty of Computing and Information Technology, King Abdulaziz University, Jeddah 21589, Saudi Arabia; thasanin@kau.edu.sa; 2 Department of Artificial Intelligence, G.H Raisoni College of Engineering, Nagpur 440016, India; pravinrk88@yahoo.com; 3 Department of Electronics and Communication Engineering, Panimalar Engineering College, Chennai 600123, India; hari13prasath@gmail.com; 4 Department of Computer Science, Cardiff Metropolitan University, Cardiff CF5 2YB, UK; sssengar@cardiffmet.ac.uk; 5 Department of Computer Science, Kebri Dehar University, Kebri Dehar 001, Ethiopia; shitharths@kdu.edu.et; 6 School of Computer Engineering, KIIT Deemed to Be University, Bhubaneswar 751024, India

The journal retracts the article titled “Exploration of Despair Eccentricities Based on Scale Metrics with Feature Sampling Using a Deep Learning Algorithm” [1], cited above.

Following publication, concerns were brought to the attention of the Editorial Office regarding the usage of unusual phrases and the relevance of a number of citations presented in this article [1].

Adhering to our standard procedure, the Editorial Office and Editorial Board conducted an investigation, which confirmed the presence of a number of unusual phrases and citations that do not confirm with MDPI’s citation policy (https://www.mdpi.com/ethics#_bookmark20), as well as a number of concerns relating to the accuracy of the figures and the overall validity of the findings. Following discussions with the authors, the Editorial Board have lost confidence in the overall findings and have decided to retract this article [1], as per MDPI’s retraction policy (https://www.mdpi.com/ethics#_bookmark30).

This retraction was approved by the Editor-in-Chief of the journal *Diagnostics*.

Suresh Chandra Satapathy, Sandeep Singh Sengar, Hariprasath Manoharan, Shitharth Selvarajan, and Pravin R. Kshirsagar disagree with this retraction. The remaining author did not provide a comment on this decision.
